# C-reactive protein – My perspective on its first half century, 1930-1982

**DOI:** 10.3389/fimmu.2023.1150103

**Published:** 2023-03-02

**Authors:** Irving Kushner

**Affiliations:** Department of Medicine, Case Western Reserve University at MetroHealth Medical Center, Cleveland, OH, United States

**Keywords:** C-reactive protein, acute phase response, complement, phagocytosis, Rockefeller Institute

## Abstract

C-reactive protein (CRP) was discovered in 1930 in the sera of patients during the acute phase of pneumococcal pneumonia and was so named because it bound to the C-polysaccharide of the pneumococcal cell wall. During the next half century many questions raised by this discovery were answered. Phosphorylcholine was found to be the moiety of the C-polysaccharide to which CRP bound. The molecular structure of CRP was elucidated: five identical subunits arranged in cyclic symmetry, giving rise to the term pentraxin. Initially felt to be not normally present in the blood, CRP was found to be a component of normal serum in trace amounts. Its site of origin was determined to be the hepatocyte. It became clear that the presumed humoral mediator responsible for CRP induction was of leukocytic origin. Binding of CRP to its ligand activated the complement system, one of the important effector mechanisms of innate immunity. CRP was found to stimulate phagocytosis of some bacterial species *via* binding to Fc receptors and was found to be protective *in vivo* against the pneumococcus in mice. It appeared likely that a related function of CRP was clearance of necrotic tissue. CRP was recognized as being a highly evolutionary conserved molecule. Its discovery during the acute phase of pneumococcal pneumonia led to its being dubbed an acute phase protein. What we today call “the acute phase response”, refers to the large number of behavioral, physiologic, biochemical, and nutritional changes that occur during inflammatory states.

## Introduction

In 1958, I started my career as a research fellow at what was then called Metropolitan General Hospital, a major teaching hospital of the Case Western Reserve University School of Medicine in Cleveland. Working under Melvin Kaplan, co-developer of the fluorescent antibody technique, I was assigned the task, employing that technique, of determining the site of production of Cx-reactive protein, as rabbit C-reactive protein (CRP) was known at the time. (We failed.) Over the ensuing years I observed the gradual accretion of knowledge about CRP, culminating in the landmark meeting under the auspices of the New York Academy of Sciences in June 1982 described below. I will focus here on what I regard as the high points of our understanding, as they evolved. This personal review does not attempt to be comprehensive or all-inclusive. I apologize to those colleagues whose work, often seminal, I have omitted.

## The discovery of C-reactive protein at The Rockefeller Institute

The Rockefeller Institute for Medical Research was established in 1906, in an era when pneumonia was a leading cause of death in the United States. When the bacteriologist Oswald Avery joined the Institute in 1913, a considerable effort in his lab was directed to understanding pneumonia, and of its most common etiologic agent, *Streptococcus pneumoniae*, generally referred to at the time as the pneumococcus. (I remind you that it was Avery’s lab which discovered in 1944 that genes were made of DNA.) (1). CRP was discovered in the course of these studies, one might rather say blundered into.

Avery and his collaborators had found that a polysaccharide (different for each pneumococcal type) made up the pneumococcal capsule, and that it was the capsule that renders the organism virulent - resistant to phagocytosis. This finding led to the use of type-specific serum therapy, the only way to treat pneumonia until the introduction of anti-microbial drugs in the late 1930s.

Then, in 1930, as recounted by Maclyn McCarty (2), William Tillett, in the Avery lab, prepared a different polysaccharide fraction, not from the capsule but rather from the cell wall, designated the “C” fraction because it appeared to be analogous to the C polysaccharide of the hemolytic streptococcus studied by Rebecca Lancefield a few years previously. Tillett and Thomas Francis set up precipitin tests against sera from serial bleedings of pneumonia patients, presuming they would detect antibodies to the “C” fraction. But the results were completely the opposite of what they had expected. A precipitate formed when the “C” substance was mixed with sera obtained at the time of admission to the hospital and throughout the febrile period (the acute phase), then diminished in the patients who recovered, and ultimately disappeared. These findings were published in 1930 (3).

This was not how antibodies behaved. It was clear that the C-precipitation phenomenon differed from immune reactions. It took the investigators a while to digest this finding. Ultimately, they realized that they were dealing with something new, different from an antibody response. It appeared that there was a substance of some kind in acute phase sera that was C-reactive.

## The molecule itself

It wasn’t until a decade later, in 1941, that a series of papers from the Avery lab reported that the C-reactive substance was a protein; thus, a *C-reactive protein* (4–6). It is of interest that these authors stated that CRP was “not normally present in the blood”.

In the early years, the Rockefeller institute largely carried the ball on CRP studies. In 1965, Emil Gotschlich and Gerald Edelman, of the Institute, reported that CRP was composed of probably identical subunits with a molecular weight of about 21,500 Da (7). The primary structure of CRP was reported in a pair of papers from Gotschlich’s lab about a decade later (8, 9). They found that CRP had a unique sequence containing 187 amino acids in a single polypeptide chain. and a minimal molecular weight of 20,946 Da. And in 1977, Alex Osmand et al, working in Henry Gewurz’s lab at the Rush Medical College in Chicago, reported that CRP was composed of five subunits arranged in cyclic symmetry, and suggested the term *pentraxin* for such a structure (10).

## Is CRP really “not normally present in the blood”?

For several decades, it was held that CRP was not normally present in the blood. Finally, in 1972, C.O. Kindmark, of the University of Lund in Sweden, employing a more sensitive assay than had previously been employed, reported that CRP was indeed a component of normal serum (11), a finding confirmed in 1976 by David Claus in the Gewurz lab (12), and again in 1981 in Mark Pepys’ lab at the Royal Postgraduate Medical School in London (13). In the latter study, serum CRP concentrations in the healthy adults they studied were under 3 mg/L in 90% of healthy adults and under 10 mg/L in almost all.

## Where is it made?

In 1966, J. Hurlimann, working in the Jeanette Thorbecke lab at New York University, found that C (14)-amino acid incorporation *in vitro* into CRP could be demonstrated in culture fluids from liver tissue, and no other organ, in inflamed rabbits and monkeys (14). A dozen years later, the specific liver cell type responsible for CRP production in rabbits was identified as the hepatocyte by Irving Kushner employing immunoenzymatic techniques, working in Gerard Feldmann’s lab at the Hôpital Beaujon in Clichy, France (15).

## How do hepatocytes know that they should produce it?

Following acute myocardial infarction, CRP concentrations were found to rise exponentially, with a mean doubling time of 8.2 h. Levels continued to rise for nearly two days in individuals with mild infarction and over three days in those with extensive infarction (16). Studies in isolated perfused rabbit livers revealed that secretion rates of CRP from livers from inflamed animals did not increase exponentially after removal of the liver from the rabbit. Rather they continued to secrete CRP at the rate achieved at the time of removal from the living animal (17), indicating that acceleration of CRP synthesis required continuing exposure to an *in vivo* mediator, as had been presumed.

By 1982, inflammation-associated cytokines just were beginning to be identified. There was considerable interest at the time in endogenous pyrogens, soluble factors from leukocytes capable of inducing fever in rabbits (18). Ralph Kampschmidt’s lab at the Samuel Roberts Noble Foundation in Ardmore, OK found that such preparations could induce a broad array of acute phase changes, including CRP, in experimental animals, as did Don Bornstein’s lab at the SUNY Upstate Medical University in Syracuse. NY (19–22), indicating that the presumed humoral mediator responsible for CRP induction was of leukocytic origin.

## Are there other acute phase proteins?

Avery’s lab referred to CRP as “the acute phase protein”. But was CRP unique, or were there other acute phase proteins? The development of immunochemical methods in the 1960s permitted quantitation of the concentrations of individual plasma proteins. It was soon learned that the concentrations of a number of plasma proteins rose (positive acute phase proteins), while concentrations of others fell (negative acute phase proteins) following inflammatory stimuli such as surgical procedures. Noteworthy positive acute phase proteins identified at the time included α_1_-antitrypsin, α_1_-acid glycoprotein, haptoglobin and ceruloplasmin, while concentrations of albumin, transferrin, α_1_-and β-lipoprotein decreased (23, 24). A few years later, Keith McAdam and Jean Sipe at the National Cancer Center in Bethesda found that the newly discovered amyloid precursor Serum Amyloid A (SAA) was also a major acute phase protein, comparable to CRP in the magnitude of increase following stimulus (25).

## Why does CRP bind to the C polysaccharide?

John Volanakis, working in Melvin Kaplan’s lab in Cleveland and Emil Gotschlich’s lab at the Rockefeller Institute independently demonstrated that phosphorylcholine was the moiety of the C polysaccharide that CRP bound to (26, 27). While CRP was found to bind to a variety of other molecules (28), phosphorylcholine was of greatest interest. Phosphorylcholine is displayed by a variety of microorganisms, raising the possibility that CRP might protect against some infections. And it is widely distributed throughout nature as a constituent of cell membranes.

## What does CRP do?

### Promotion of inflammation by complement activation

Two laboratories, those of Volanakis and Kaplan in Cleveland, and J. Siegal and R. Rent in the Gewurz lab, in the mid-1970s reported that binding of CRP to its ligand activated the complement system *via* the classical pathway (29–32). Activation of complement, one of the important effector mechanisms of innate immunity, leads to opsonization of pathogens as well as to generation of the classical inflammatory response.

### Phagocytosis of bacteria

CRP was found to bind to some pathogenic bacteria, raising the possibility that it facilitated phagocytosis; that it might be an opsonin (33, 34) CRP was found to stimulate phagocytosis of some bacterial species (35–37). But phagocytosis requires phagocytes. Richard Mortenson and Jo Ann Duszkiewicz at Ohio State University and Karen James and B. Hansen in the Gewurz laboratory reported that CRP binds to macrophages and lymphocytes via Fc receptors (38, 39). Indeed, CRP was found to be protective in vivo against the pneumococcus by Carolyn Mold, Terry Du Clos and their collaborators, and workers in the Volanakis lab (40, 41).

### Clearance of necrotic tissue

In experimental animals in which tissue injury was induced by intramuscular injection of croton oil, Kushner and Kaplan, employing immunofluorescent methods, found that CRP localized to necrotic myofibers and no other tissue (42). Comparable CRP localization to necrotic cardiac muscle fibers was seen when myocardial infarction was induced by cardiac artery ligation (43).

These findings raised the possibility that a function of CRP might be to target necrotic tissue for removal of necrotic debris, facilitating phagocytosis of damaged cells. Phosphorylcholine is a constituent of all cell membranes, and this possibility was supported by the finding in John Volanakis’ lab that CRP bound to artificial phosphatidylcholine bilayers (44) but that alteration of the normal organization of phosphatidylcholine bilayers was required for binding of CRP to occur (45). Necrotic cells are cleared by phagocytic cells, and the role of CRP in their clearance is undoubtedly comparable to its role in phagocytosis of bacteria – necrotic cells are targeted by CRP, which in turn binds to Fc receptors on phagocytes. In addition, the activation of complement would lead to initiation of innate immunity, and generation of the classical inflammatory response locally.

## Phylogeny

CRP is a highly conserved molecule (46). Early studies from the Rockefeller lab identified CRP in sera from rabbits and monkeys (47). Before long it was also identified in several land-based vertebrate species (48) and then in the fish species Plaice (49). It turned out that CRP appeared very early in the course of evolution; a protein from the hemolymph of *Limulus polyphemus* – the Horseshoe Crab (nearly half a billion years on this planet) - was found which resembles human CRP (50).

## Clinical considerations

The original discoverers of CRP felt that it was elicited by infection with Gram positive organisms. But as early as 1933, Rachel Ash, of the Children’s Hospital in Philadelphia, employing a precipitation test with the C polysaccharide, found that infection with Gram negative organisms also gave positive tests (51). The first clinical use for determination of CRP in the blood, developed at the Rockefeller Institute by H. C. Anderson and Maclyn McCarty, employed antibodies to CRP in a capillary precipitin test as a marker of disease activity in acute rheumatic fever (52). Subsequently, hundreds of papers were published reporting the use of CRP determination to indicate the presence of an inflammatory process, estimate its severity, and monitor the course of illness in a great variety of medical conditions.

## The acute phase response

Oswald Avery had been fascinated by the appearance of CRP during the acute phase of infectious diseases. He was quoted by Rene Dubos: “Avery never discussed the C-reactive protein without turning the conversation to what he was wont to call ‘the chemistry of the host’. Although he never spelled out what he meant by that expression, he clearly had in mind all the unidentified body substances and mechanisms of a nonimmunological nature, both protective and destructive, that come into play in the course of infectious processes” (53). It soon became apparent that the acute phase response was not limited to infectious processes, but also occurred after tissue injury, such as occurs in surgical procedures and myocardial infarction. And although CRP is an “acute phase” protein, it was clear from the beginning that the acute phase response occurs in chronic diseases as well as in acute diseases, and often persists for extended periods. What Avery termed the “chemistry of the host” is today termed “the acute phase response” - the large number of behavioral, physiologic, biochemical, and nutritional changes that occur during inflammatory states (54).

## Taking stock after half a century

In 1981, Mark Pepys brought CRP to the attention of the broad biomedical community with an essay published in the Lancet, classified by them as an “Occasional Survey”, with a thorough, scholarly summary of what was known about CRP at the time (28).

The late John Volanakis, Henry Gewurz and I ran into one another at a meeting of The Central Society for Clinical Research at the Drake Hotel in Chicago in November 1979. We were aware that the late 1970s had seen increasing interest in CRP and the acute phase response and concluded that the time had come to organize an international meeting about CRP, SAA and the acute phase response in general. Under the auspices of the New York Academy of Sciences, that meeting, entitled “C-Reactive Protein and the Plasma Protein Response to Tissue Injury”, was held at the Barbizon-Plaza Plaza Hotel in New York City on September 21-23, 1981 (55). (The original projected title, “C-Reactive Protein and the Acute Phase Response”, was rejected because it was felt that very few scientists were familiar with the term “Acute Phase Response”, and inflammation was generally defined at the time as the response to tissue injury.) Thirty-six papers were presented at this meeting, and there were 26 posters.

The field has taken off since then.

## Author contributions

The author confirms being the sole contributor of this work and has approved it for publication.
